# RTK-RAS pathway mutation is enriched in myeloid sarcoma

**DOI:** 10.1038/s41408-018-0083-6

**Published:** 2018-05-23

**Authors:** Mihong Choi, Yoon Kyung Jeon, Choong-Hyun Sun, Hong-Seok Yun, Junshik Hong, Dong-Yeop Shin, Inho Kim, Sung-Soo Yoon, Youngil Koh

**Affiliations:** 1 Department of Internal Medicine, Seoul National University Hospital, Seoul, Korea; 2 Department of Pathology, Seoul National University Hospital, Seoul, Korea; 3 Development Group, Samsung SDS, Seoul, Korea; 4 Department of Bioinformatics, Seoul National University Hospital, Seoul, Korea

Myeloid sarcoma (MS), or granulocytic sarcoma, is a rare extramedullary tumor of immature myeloid cells. MS may present simultaneously with or during the course of acute myeloid leukemia (AML), myelodysplastic syndrome (MDS), or any forms of myeloproliferative neoplasms (MPN). Less commonly, it is detected as an isolated form without bone marrow (BM) involvement. Although the prognosis of MS has not been well examined due to the rarity of this disorder, it is known to be refractory to standard therapies of AML and is generally associated with a poor outcome^1^. Furthermore, it has been noted that patients with myeloid sarcoma have a predisposition to extramedullary relapses^2^.

Recently, immune checkpoint blockade with ipilimumab was shown to induce complete remission in four patients with extramedullary relapse after allogeneic hematopoietic stem cell transplantation (HSCT) for AML, which lasted for more than 1 year in two of them^3^. This was an intriguing therapeutic breakthrough in that extramedullary myeloid leukemia, which had hitherto been an area of unmet medical need for being unamenable to standard treatment, was highly susceptible to immuno-oncology drugs.

In light of this distinct biologic behavior of MS compared to that of conventional AML, namely, homing and clustering outside the hematopoietic system, being responsive to immune checkpoint inhibitors while refractory to conventional chemotherapeutic agents used in AML, we hypothesized that MS could share some of the genetic abnormalities commonly found in solid tumors demonstrating features mimicking them. The aim of this study was to explore this using a more expanded panel of cancer genes, which are not necessarily restricted to known AML-associated genes, to gain insight into the molecular pathogenesis of MS and to identify potential drug targets.

We retrospectively identified and collected clinical data of 62 patients with a diagnosis of MS made between March 2003 and May 2016 at Seoul National University Hospital (SNUH). Of these, 13 patients went through planned panel sequencing of 83 genes (Figure S1). The study protocol was reviewed and approved by the Institutional Review Board of SNUH.

Genomic DNA was isolated from formalin-fixed paraffin-embedded (FFPE) tumor tissue blocks using the QIAamp DNA Mini Kit (Qiagen, Mancheester, UK), and the qualified DNA samples were captured and sequenced with SureSelect (Agilent, Inc., USA) following the manufacturer’s instructions. The targeted 83 cancer genes were concentrated more on well-known oncogenes reported in the Catalog of Somatic Mutations in Cancer database than on relatively unknown genes whose functional effects are currently in question and included the coding exons of 72 genes for the detection of single nucleotide variants (SNVs), insertion/deletions (indels), and copy number variations (CNVs), and some introns for 5 genes for the detection of gene fusions. The mean coverage of all the samples was 673 × (range 33–1506). (see Supplementary Methods for details).

Sixty-two patients with a clinical and/or pathologic diagnosis of MS were included in our clinical analysis, whose median age at presentation was 46 years old (range 18–83), and the female-to-male ratio was 1.06. These MS cases presented most commonly with a concurrent initial diagnosis of AML (33.9%) followed by a relapse or persistence of marrow disease (22.6%), and so on. Except for de novo MS, all cases were accompanied by AML (Table S1). Results of the clinical analysis are depicted in the Supplementary Results.

Table 1 and Table S3 summarize the clinical and/or mutational data by case in our series. All 13 sequenced cases had at least one well-known oncogenic mutation, and more than one mutation was found in four patients, with all cases positive for the *IDH2* and/or *NPM1* mutation in the current study exhibiting another co-mutation. Although the number of sequenced cases in this series was too small to test for a certain trend, patients with normal cytogenetics from their BM tended to have more known point mutations in MS than their counterparts (*p* = .066). In contrast, age seemed to have no association with the number of driver mutations in the MS samples (*p* = .178), whereas it correlated with the number of mutations in the reported data of AML^4^.

**Table 1:** Clinical and mutational profiles of sequenced cases

Case	Sex/age	BM diagnosis, FAB classification	BM cytogenetics	Presentation	Involved sites	Mean target coverage	Known somatic mutations (VAF)	Novel non-synonymous variants of unknown significance (VAF)
1	F/50	AML with MDS-related change	45,XX,del(5)(q?22q33),-7[13]/46,XX[7]	Isolated relapse	Left and right breasts, right ventricle, left axilla, both flank, inguinal area, and stomach antrum	1447.59	GNAQ_c.286A>T_p.T96S (5.7) IDH2_c.419G>A_p.R140Q (43.9) NRAS_c.35G>A_p.G12D (44.1)	
2	F/41	AML M2	46,XX,t(8;21)(q22;q22) [10]	Isolated relapse	Right frontal lobe, scalp, both ovaries, neck LNs, bones, mesentery	45.91	SKT11_c.1190C>T_p.A397V (22)	ARID1A_c.757C>G_p.P253A (29) BRAF_start_gained (13.1) NOTCH1_c.1843G>A_p.G615R (21.8)
3	F/34	AML M5a	46,XX,t(11;19)(q23;p13.1)[17]/46,XX[3]	Isolated relapse	Rt. anterior chest, cervical and mediastinal LNs	473.41	NRAS_c.182A>T_p.Q61L (34.1)	NOTCH1_c.3350A>G_p.Q1117R (50)
4	F/47	AML M2	NK, FLT3+/NPM1+	Isolated relapse	Rt. anterior chest skin, Rt. axilla, Lt. scapula, skull base, leptomeningeal seeding	1506.30	FLT3_c.1800_1801insTAGTATAAGTATAAGAGACTT_p.599D_600LinsDFREYEYD (77.9) IDH2_c.419G>A_p.R140Q (44.9) NPM1_ c.860_861insTCTG_ p.286L_287Wfs (37.6)	KDR_splice_site (15.8) PTCH2_ c.2668T>C_ p.Y890H (49.6)
5	M/22	AML M5a	47,XY,+8[3]/46,XY[4]	Isolated relapse	Skin	1253.65	KRAS_c.38G>A_p.G13D (48.7) PTEN_deletion (0, -1.99)	AURKB_c.487C>G_p.L163V (49.9) MTOR_ c.1919A>G_ p.H640R (64) 0NF1_c.7774C>A_p.H2592N (46.2)
6	M/70	AML transformed from CMMoL	NK, no point mutations	Concurrent	Anterior chest	152.40	ERBB2_c.3149C>T_p.S1050L (39.4) NRAS_c.181C>A_p.Q61K (52.6)	BRCA1_c.4792T>C_p.S1598P (49.4) BRCA2_c.1342C>A_p.R448S (5.9) KDR_splice_site (19) NF1_c.668G>T_p.W223L (7) PDGFRA_c.2518G>A_p.A840T (16.9)
7	F/49	AML M2	46,XX,t(8;21)(q22;q22)[20]	Concurrent	Right breast, cardiophrenic and retrosternal LNs, paravertebral TLS	33.66	KIT_c.2447A>T_p.D816V (60)	ATM_c.533C>T_p.P178L (21.4) BRCA2_c.6376T>C_p.C2126R (63.8) CSF1R_c.263G>C_p.G88A (45.2) JAK3_splice_site (61.1)
8	F/33	AML M4	NK, no point mutations	Concurrent	Nasopharynx	1231.71	GNAQ_c.286A>T_p.T96S (5.9)	ATRX_c.1492A>G_p.R498G (47.1)
9	F/60	AML unclassified	46,XX,16qh+[20], NPM1+	Concurrent	Cervical and portocaval LNs, bones	512.87	IDH2_c.419G>A_p.R140Q (29.4) NPM1_ c.860_861insTCTG_ p.286L_287Trpfs (28.8) NRAS_c.37G>C_p.G13R (21.9)	APC_c.1984C>A_p.L662I (47.1) BRAF_start_gained (43.9) ROS1_splice_site (47.6)
10	F/60	AML unclassified	46,XX,t(6;9)(p22;q34), FLT3ITD+	Concurrent	Left lower leg (skin), subcutaneous and intramuscular nodules, bones, lungs	1183.80	FLT3_c.1800_1801insTAGTATAAGTATAAGAGACTT_p.599D_600LinsDFRQYQYD (22.2)	FGFR1_splice_site (52.9) FLT3_ c.773C>T_p.P258L (50.1)
11	M/47	AML M4	45,X-Y,t(8;21)(q22;q22),9qh-[20]	Marrow relapse	Left anterior chest wall	231.44	KIT_c.2447A>T_p.D816V (65.8)	APC_c.2651C>T_p.A884V (66) BRCA2_c.6715G>A_p.E2239K (47.8) BRCA2_c.7096C>G_p.L2366V (49.8) RB1_c.2035A>G_p.I679V (49.5) ROS1_c.6763T>C_p.S2255P (40.4) ROS1_c.6764C>T_p.S2255L (40.2) SMO_c.1068G>T_p.K356N (23.5)
12	M/60	AML unclassified	46,XY,t(1;7)(q21;p22)[3]/46,sl,t(4;21)(q21;q22)[3]/49,sdl1,+3,+8,+21[9]/46,XY[6]	Marrow relapse	Left humerus (pathologic fracture)	553.88	JAK2_c.1849G>T_p.V617F (72.4)	
13	M/11	AML M4	46,XY,t(8;15)(q22;q26),inv(9)(p11q13),del(9)(q22)[10]	Concurrent	Gingiva, scrotum (sequential)	124.07	APC_c.904C>T_p.R302* (55) FBXW7_c.1634A>G_p.Y545C (95.9) KRAS_c.35G>A_p.G12N (43.5) PIK3CA_c.1634A>C_p.Q545A (38.2) RET_c.2053G>A_p.V685I (46.9) TP53_c.856G>A_p.E286K (70.3)	ALK_c.4433T>C_p.M1478T (5.1) APC_c.4461_4462delT_p.1486T_1487Lfs (50.6) NOTCH1_splice_site (7.7) SMO_c.1769G>C_p.S590T (31.4)

*BM* bone marrow, *AML* acute myeloid leukemia, *FAB* French-American-British, *VAF* variant allele frequency, *MDS* myelodysplastic syndrome, *LN* lymph node, *NK* normal karyotype, *CMMoL* chronic monomyelocytic leukemia

Strikingly, most of them (11 out of 13 cases) had a mutation in the genes of the receptor tyrosine kinase (RTK)-RAS pathway. *NRAS* was the most frequent genetic alterations among these, affecting four cases. *FLT3* ITD, *KIT*, and *KRAS* each were found in two patients, whereas *ERBB2, JAK2, PIK3CA*, and *RET* each were identified in one case. Of the affected genes not grouped as the RTK-RAS pathway, the *IDH2* R140Q mutation was reported in three cases, and the *NPM1* mutation was found in two cases, which was consistent with their marrow findings. Interestingly, *GNAQ* T96S was reported in two cases with an allele frequency of 5.7 and 5.9, respectively. The functional effect of this mutations is yet to be known, although it has been previously identified in sequencing studies on melanoma^5^ and pancreatic adenocarcinoma^6^, and computationally predicted to be deleterious by LRT^7^ and FATHMM^8^.

CNVs were reliably analyzed in 5 samples, where the mean target coverage was approximated to be 1000–1500 × : #1, #4, #5, #8, and #10. Of these, only case #5 was remarkable for *PTEN* deletion. No known gene fusion was found among those 13 cases.

Because previous studies have already disclosed the comprehensive mutational landscape of AML, we compared the mutational frequency of the genes sequenced in this series with that from the reported data of AML^4,9^. As noted above, most of the driver mutations in MS occurred in genes of the RTK-RAS pathway, and their mutational frequency as a group was 84.6%, which was significantly greater than that of 43.1 and 54.6% in AML, as reported from whole genome and whole exome sequencing in the Cancer Genome Atlas and extensive target sequencing involving more than 1500 AML patients, respectively (*p* = .007 and *p* = .046, respectively; Table 2)

**Table 2:** Comparison of mutational frequency in MS with reported data

	MS series (*n* = 13)	AML (*n* = 188)	*p*	OR (95% CI)	AML (*n* = 1540)	*p*	OR (95% CI)
*NRAS*	4 (30.8%)	15 (8.0%)	0.024	5.06 (1.017–21.003)	270 (17.5%)	0.263	2.09 (0.467–7.553)
*FLT3* ITD	2 (15.4%)	54 (28.7%)	0.522	0.45 (0.047–2.178)	521 (33.8%)	0.240	.36 (0.038–1.639)
*KIT*	2 (15.4%)	7 (3.7%)	0.108	4.64 (0.423–28.704)	66 (4.3%)	0.108	4.05 (0.428–19.139)
*KRAS*	2 (15.4%)	8 (4.3%)	0.130	4.04 (0.375–24.053)	79 (6.8%)	0.145	3.36 (0.356–15.783)
*JAK2*	1 (7.7%)	1 (0.5%)	0.126	15.01 (0.183–1215.0)	11 (0.7%)	0.096	11.52 (0.249–92.017)
*ERBB2*	1 (7.7%)	0	0.065	NA (0.371–NA)	NA	NA	NA
*PIK3CA*	1 (7.7%)	0	0.065	NA (0.371–NA)	NA	NA	NA
*RET*	1 (7.7%)	0	0.065	NA (0.371–NA)	NA	NA	NA
RTK-RAS genes	11 (84.6%)	81 (43.1%)	0.007	7.20 (1.510–68.768)	841 (54.6%)	0.046	4.57 (0.992–42.492)
*GNAQ*	2 (15.4%)	0	0.004	NA (2.839–NA)	NA	NA	NA
*STK11*	1 (7.7%)	0	0.065	NA (0.371–NA)	NA	NA	NA
*IDH2*	3 (23.1%)	19 (10.1%)	0.157	2.65 (0.432–11.569)	108 (7.5%)	0.060	3.97 (0.692–15.730)
*NPM1*	2 (15.4%)	52 (27.7%)	0.520	0.477 (0.050–2.297)	440 (28.6%)	0.371	0.455 (0.049–2.096)

*MS* myeloid sarcoma, *AML* acute myeloid leukemia, *OR* odds ratio, *CI* confidence interval, *ITD* internal tandem duplication, *NA* not applicable, *RTK-RAS genes* receptor tyrosine kinase-RAS pathway genes

This would hint at a likely pathophysiology of MS in part. Being subclonal, mutation of the RTK-RAS signaling genes is inferred as a late event in leukemogenesis of AML^4,10^. Likewise, MS has a good chance of occurring late in AML evolution, acquiring additional mutations in the process that potentially explain the unusual tropism of the myeloid blasts for extramedullary tissues. It also has an important therapeutic implication because biochemical inhibition of oncogenic Ras signaling is being actively studied with FLT3 inhibitors being at the forefront^11^ closely followed by BLU-285, a potent and selective inhibitor of the exon 17 mutant KIT kinase^12^. On the other hand, clonal mutation including *IDH2* R140Q is retained in MS, so that molecular targeted therapy against these early lesions is expected to be effective on MS as well. Furthermore, if multiple mutations are gained to engender myeloid blasts to home outside the BM, this high mutational burden of MS can be predictive of its responsiveness to immunotherapy^13^, as is the case with ipilimumab for extramedullary relapse of AML^3^.

There were two previous NGS studies of MS comparable to this study: Li et al. and Pastoret et al. reported on the results of targeted sequencing of 21 and 28 genes from 6 and 14 MS cases, respectively. Genetic abnormalities were found in various AML-associated genes encoding tyrosine kinases (*FLT3, KIT*, and *KRAS*), tumor suppressors (*WT1* and *TP53*), epigenetic modifiers (*TET2* and *ASXL1*), spliceosome proteins (*SF3B1* and *SRSF2*), and transcription factors (*RUNX1*)^14,15^. Although the current analysis partially reproduced these results, both of the prior studies used panels consisting of a limited number of genes rendering their results inconclusive to examine whether a certain oncogenic pathway is affected in MS. In addition, novel variants discovered from panel sequencing can either be a pathogenic mutation or neutral variation, for which we restricted our analysis to well-known variants.

Our study nevertheless has several limitations. First, this is a retrospective study with unavoidable selection bias. Second, as we extracted DNA from FFPE, artifacts caused by fixation and storage cannot be ruled out, and the CNV analysis was unreliable for most samples presumably reflecting this. Third, although we tested an expanded set of genes compared to earlier studies, genes not included in our panel could have an important implication. In addition, we did not assess the functional consequence of identified mutations. Furthermore, the referenced data of AML included AML with MS as well as AML without MS. We believe, however, that this would have reinforced our point, if the mutational profile of MS had been compared only with that of AML without MS. Lastly, the small sample size of our analysis undermines the statistical power, although these few cases consistently demonstrated RTK-RAS enrichment.

In summary, the pattern of molecular derangements in MS was generally consistent with that in AML, but MS was apparently more enriched with mutations of the RTK-RAS pathway genes, sharing genetic commonalities with solid tumors than with AML. Future studies are warranted to elucidate their therapeutic and prognostic implications as well as the detailed molecular mechanism underlying their distinct phenotypic expression.

## Electronic supplementary material

Supplementarly information
