# Supplementary material for: RTK-RAS pathway mutation is enriched in myeloid sarcoma
Source: Blood Cancer J. 2018 May 23;8(5):43. doi: 10.1038/s41408-018-0083-6 (PMC5964090; doi:10.1038/s41408-018-0083-6)
Supplement: Supplementary file 1 — Supplementarly information [file 41408_2018_83_MOESM1_ESM.pdf]

Supplementary Information

1. Supplementary methods

Patient selection and data gathering

Targeted sequencing

Sequencing data analysis

Statistical method

2. Supplementary results

Baseline characteristics of patients

Immunophenotypic profile of MS

Survival of patients with MS

Additional mutational profile of MS

3. Supplementary tables

Table S1. Baseline characteristics

Table S2. Immunophenotypic profile of MS

Table S3. Known somatic mutations among reported variants with allele frequency ≥5%

4. Supplementary figures

Figure S1. Patients selection and flow of analysis

Figure S2. Immunophenotypic profile of MS

Figure S3. Survival curves of patients with MS by their presentation

5. References

1. Supplementary methods

Patient selection and data gathering

We retrospectively identified and collected clinical data from 62 patients whose ages were 18 years or older with a diagnosis of MS made between March 2003 and May 2016 at Seoul National University Hospital. These include 14 clinically-diagnosed MS cases without histologic confirmation, whose clinical features strongly support the diagnosis of MS rather than an alternative etiology. The clinical data collected and analyzed consist of age, sex, temporal relationship between MS presentation and marrow disease, diagnosis and characteristics of marrow disease, hemogram at the point of MS diagnosis, immunophenotypic profile of MS, and survival, if available.

Of these, 13 patients went through the planned panel sequencing of 83 genes, after excluding 14 cases with clinically-diagnosed MS without histologic confirmation, 10 patients with de novo MS without marrow involvement, and 25 cases whose specimen contained an insufficient tumor or inadequate DNA yield for the sequencing (Supplementary Figure 1).

Targeted sequencing

Genomic DNA was isolated from formalin-fixed paraffin-embedded (FFPE) tumor tissue blocks prepared as 5 ㎛ thick sections with the QIAamp DNA Mini Kit (Qiagen, Mancheester, UK). Extracted DNA was quantified with a Qubit fluorometric quantitation (Life Technologies, Grand Island, NY, USA), and NanoDrop ND-1000 spectrophotometer (NanoDrop Technologies, Wilmington, USA). The mean concentration of the extracted DNA was 116.19 ng/㎕ (range: 9.38-464.00 ng/㎕), and the measured 260/280 purity was from 1.75 to 1.98.

The qualified DNA samples were captured and sequenced with SureSelect (Agilent, Inc., USA) following the manufacturer's instructions. Briefly, they were randomly fragmented by a Covaris sonicator (Covaris, Woburn, MA), ligated with adapters, purified, hybridized, and amplified to construct a captured library, which was then loaded on to

the Illumina HiSeq2500 (TheragenEtex Bio Institute, Suwon, Korea). Raw image files were processed by HCS 1.4.8 for base-calling with the default parameters, and the sequences of each individual were generated as 101 bp paired-end reads.

The targeted 83 cancer genes included the coding exons of the following 72 genes for the detection of single nucleotide variants (SNVs), insertion/deletion (indels), and copy number variations (CNVs): *ABL1, AKT1, AKT2, AKT3, ALK, APC, ARID1A, ARID1B, ARID2, ATM, AURKA, AURKB, BCL2, BRAF, BRCA1, BRCA2, CDH1, CDK4, CDK6, CDKN2A, CSF1R, CTNNB1, DDR2, EGFR, EPHB4, ERBB2, ERBB3, ERBB4, EWSR1, EZH2, FBXW7, FGFR1, FGFR2, FGFR3, FLT3, GNA11, GNAS, GNAQ, HNF1A, HRAS, IDH1, IDH2, IGF1R, ITK, JAK1, JAK2, JAK3, KDR, KIT, KRAS, MDM2, MET, MLH1, MPL, MTOR , NF1, NOTCH1, NPM1, NRAS, NTRK1, PDGFRA, PDGFRB , PIK3CA, PIK3R1, PTCH1, PTCH2, PTEN, PTPN11, RB1, RET, ROS1, SMAD4, SMARCB1, SMO, SRC, STK11, SYK, TERT, TMPRSS2, TOP1, TP53*, and *VHL*. Additionally, some introns of the following 5 genes were covered for the detection of gene fusions: *ALK, RET, ROS1, EWSR1*, and *TMPRSS2*. The mean coverage of all the samples was 718x (range 33-1506).

Sequencing data analysis

Sequence reads were aligned to the human genome 19 reference using BWA-MEM,[1] after which GATK Best Practice of Broad Institute was used for the removal of duplications, local realignment, and recalibration.[2]

After filtering for germline polymorphisms and false positives, SNVs with a variant allele frequency (VAF) ≥5% and Indels with a VAF ≥10% were selected as the final results. SNVs and Indels were detected with the ensemble method integrating three open source callers: the UnifiedGenotyler[3], LoFreq[4], SNVer[5], and SamsungSDS's in-house callers.

CNVs were analyzed using the depth of coverage for each target region between the tumor and preprocessed normal data. To calculate the absolute copy number, tumor purity and ploidy were estimated from a statistical model using the log2 ratio values and the SNV VAF values. As a cut-off value, CN ≥7 and CN =0 were used for amplification and homo-

deletion, respectively. For translocation detection, a paired-end mapping analysis and a split-alignment analysis were applied. All discordant read-pairs with an abnormal insert-size or orientation were screened, and soft-clipping information of the split-reads was investigated as evidence of genomic rearrangements. The cut-off value of the confident translocations was a split-read support count ≥3. CNVs and translocation were discovered with in-house callers developed by SamsungSDS.

Statistical method

Overall survival (OS) was calculated as the time from diagnosis of MS to death from any cause, and survival curves were estimated and compared by the Kaplan-Meier method and log-rank test, respectively. Patients were censored at the time of the last visit until May 1, 2017.

Mutational frequency of the MS cohort was compared to that from the referenced AML data by Fisher's exact test. Number of known point mutations was tested for their association with the presence or absence of cytogenetic abnormalities using the T-test and with age by the Spearman rank correlation analysis.

2. Supplementary results

Baseline characteristics of the patients

Sixty-two patients with clinical and/or pathologic diagnosis of MS were included in our clinical analysis, whose median age at presentation was 46 years (range 18-83 years), and the female-to-male ratio was 1.06 (32/30). These MS cases presented most commonly with a concurrent initial diagnosis of marrow disease (33.9%) followed by relapse or persistence of marrow disease (22.6%), as an isolated relapse after treatment of the marrow disorder (21.0%), *de novo* MS (16.1%), or antedating diagnosis of marrow disease (6.5%), in decreasing order of frequency.

Except those without a history of marrow involvement, all cases with marrow involvement were accompanied by AML, whether it be secondary to MPN or MDS (19.0%) or *de novo* AML (63.5%). M4 and M2 were the most common type of AML according to the French-American-British classification, representing 19.4% and 16.1% of the cases, respectively. In addition, the normal and complex karyotype were most commonly reported in their marrow cytogenetic analyses, accounting for 41.9% and 21.0% of the patients, respectively. These baseline characteristics are summarized in Supplementary Table 1.

Immunophenotypic profile of MS

As expected, myeloperoxidase (MPO) was the most commonly used item with 75% of the cases tested and a sensitivity of 91.5% among them. Although CD43, CD117, lysozyme, and CD68 were tested less frequently in about 50%, 40%, 25%, and 15% of the tissues, their sensitivities were as high as 96.6%, 76%, 80.0%, and 80.0%, respectively. The immunophenotypic profiles of MS are specified in Supplementary Table 2 and Supplementary Figure 2.

Survival of patients with MS

Survival of MS patients differed by their setting of presentation, i.e., patients with *de novo* MS without marrow

involvement exhibited the longest survival followed by the group with MS antedating or following AML, concurrent MS with newly-diagnosed AML, and MS developed with persistent or relapsed AML, with a 1 year survival rate of 80.0%, 63.2%, 47.6%, and 28.6%, respectively (log-rank *p* = .001; Supplementary Figure 3A).

When the study population was grouped by presence or absence of marrow involvement, regardless of whether it coexisted with MS development, the patients with marrow disease had a significantly shorter survival than their counterpart with a 1 year survival rate of 80.0% and 47.6%, respectively (log-rank *p* = .014; Supplementary Figure 3B).

Additional mutational profile of MS

Previously-unreported non-synonymous variants were reported more frequently than known variants within cancer-related genes targeted in our MS cohort. Of these, *BRAF* start-gained was identified repeatedly in 2 cases, and its clinical implication is currently in question (Table 1).

3. Supplementary tables

Table S1. Baseline characteristics

Variables		Frequency (%)
**Age in years, median (range)**		46 (18-83)
**Sex**	**Male**	30 (48.4%)
	**Female**	32 (51.6%)
**Presentation of MS**	**Concurrent with marrow disease**	21 (33.9%)
	**With relapse or persistence of marrow disease**	14 (22.6%)
	**As an isolated relapse after marrow disease**	13 (21.0%)
	**Antedating marrow disease**	4 (6.5%)
	**De novo MS**	10 (16.1%)
**BM diagnosis**	**AML**	40 (63.5%)
	**Secondary AML with previous marrow disorders**	12 (19.0%)
	**De novo MS**	10 (15.9%)
**AML FAB classification**	**M0**	1 (1.6%)
	**M1**	7 (11.3%)
	**M2**	10 (16.1%)
	**M3**	2 (3.2%)
	**M4**	12 (19.4%)
	**M5**	4 (6.5%)
	**Unclassified or unknown**	26 (41.9%)
**Marrow cytogenetics**	**Normal**	26 (41.9%)
	**t(8;21)**	9 (14.5%)
	**inv(16) or t(16;16)**	4 (6.5%)
	**MLL t(11q23;x)**	3 (4.8%)
	**t(6;9)**	1 (1.6%)
	**t(15:17)**	1 (1.6%)
	**del(5q)**	1 (1.6%)
	**del(20q)**	1 (1.6%)
	**Complex karyotype**	13 (21.0%)
	**Unknown**	3 (4.8%)

**WBC count in /μℓ, median (range)**	5530 (350-307370)
**Hemoglobin in g/dℓ, median (range)**	10.8 (6.5-15.4)
**Platelet count in x10^3/μℓ, median (range)**	115 (3-548)
**Lactate dehydrogenage in IU/L, median (range)**	289 (95-1026)

Abbreviations: MS, myeloid sarcoma; BM, bone marrow; AML, acute myeloid leukemia; FAB, French-American-British; WBC, white blood cell

Table S2. Immunophenotypic profile of MS

		Positive	Weak or focal positive	Negative	Unknown	Testing frequency (%)	Sensitivity (%)
**Items**	**MPO**	30 (48.4%)	13 (21.0%)	4 (6.5%)	15 (24.2%)	75.81	91.49
	**Lysozyme**	6 (9.7%)	6 (9.7%)	3 (4.8%)	47 (75.8%)	24.19	80.00
	**TdT**		6 (9.7%)	17 (27.4%)	39 (62.9%)	37.10	26.09
	**CD3**		15 (24.2%)	23 (37.1%)	24 (38.7%)	61.29	39.47
	**CD10**		1 (1.6%)	6 (9.7%)	55 (88.7%)	11.29	14.29
	**CD20 (L26)**		12 (19.4%)	27 (43.5%)	23 (37.1%)	62.90	30.77
	**CD34**	7 (11.3%)	3 (4.8%)	17 (27.4%)	35 (56.5%)	43.55	37.04
	**CD43**	25 (40.3%)	3 (4.8%)	1 (1.6%)	33 (53.2%)	46.77	96.55
	**CD45 (LCA)**		10 (16.1%)	3 (4.8%)	49 (79.0%)	20.97	76.92
	**CD56**		3 (4.8%)	7 (11.3%)	52 (83.9%)	16.13	30.00
	**CD68**	1 (1.6%)	7 (11.3%)	2 (3.2%)	52 (83.9%)	16.13	80.00
	**CD79a**	1 (1.6%)	1 (1.6%)	6 (9.7%)	54 (87.1%)	12.90	25.00
	**CD117**	12 (19.4%)	7 (11.3%)	6 (9.7%)	37 (59.7%)	40.32	76.00
	**CD123**	1 (1.6%)	2 (3.2%)	3 (4.8%)	56 (90.3%)	9.68	50.00
	**CD138**		1 (1.6%)	2 (3.2%)	59 (95.2%)	4.84	33.33
	**EBV**		1 (1.6%)	5 (8.1%)	56 (90.3%)	9.68	16.67
	**Ki-67**	median 50.00 (range, 3-85)			25 (40.3%)		

Abbreviations: MS, myeloid sarcoma; MPO, myeloperoxidase; LCA, leukocyte common antigen; EBV, Ebstein-barr virus; SD, standard deviation

Table S3. Known somatic mutations among reported variants with allele frequency ≥5%

Case	Mean coverage	*ERBB2*	*FLT3*	*GNAQ*	*IDH2*	*JAK2*	*KIT*	*KRAS*	*NPM1*	*NRAS*	*PIK3CA*	*RET*	*STK11*	*TP53*
1	1447.59			★T96S (5.7)	★ R140Q (43.9)					☆ G12D (44.1)				
2	45.91												☆ A397V (22)	
3	473.41									☆ Q61L (34.1)				
4	1506.30		☆ITD (77.9)		★ R140Q (44.9)				★ W287fs* (37.6)					
5	1253.65							☆G13D (48.7)						
6	152.40	☆ S1050L (39.4)								☆ Q61K (52.6)				
7	33.66						★ D816V (60)							
8	1231.71			★T96S (5.9)										
9	512.87				★ R140Q (29.4)				★ W287fs* (28.8)	☆ G13R (21.9)				
10	1183.80		☆ITD (22.2)											
11	231.44						★ D816V (65.8)							
12	553.88					☆ V617F (72.4)								
13	124.06							☆G12D (43.5)			☆E545A (38.2)	☆ V685I (46.9)		☆ E286K (70.3)

4. Supplementary figures

Figure S1. Patients selection and flow of analysis

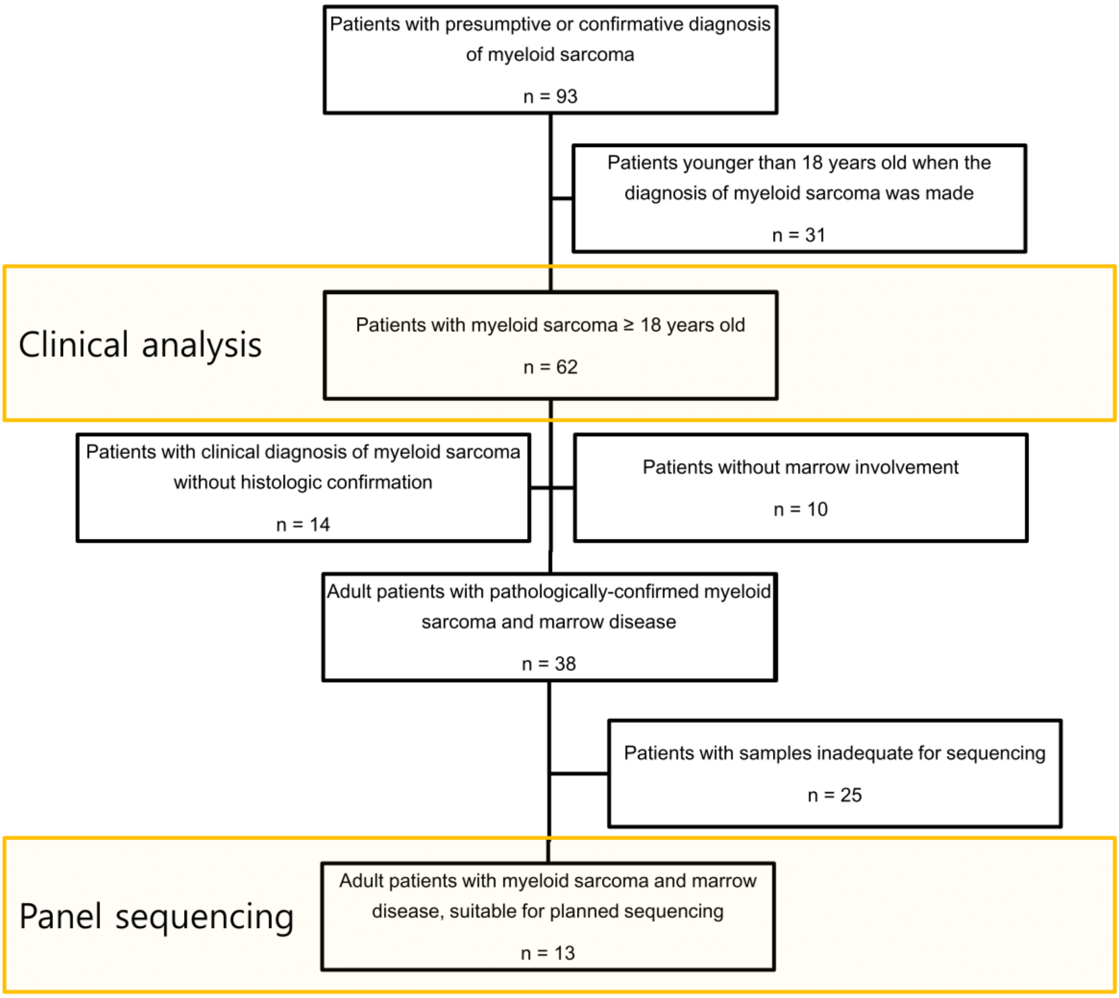

Figure S2. Immunophenotypic profile of MS

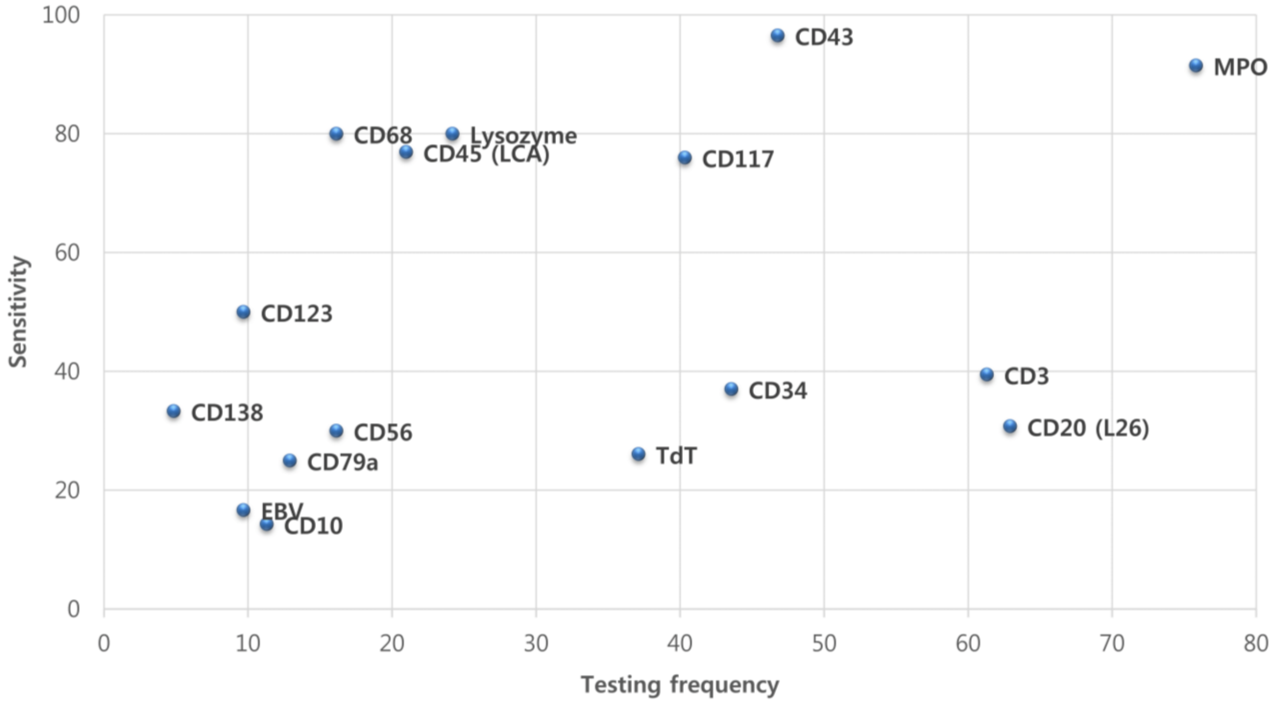

Figure S3. Survival curves of patients with MS by their presentation

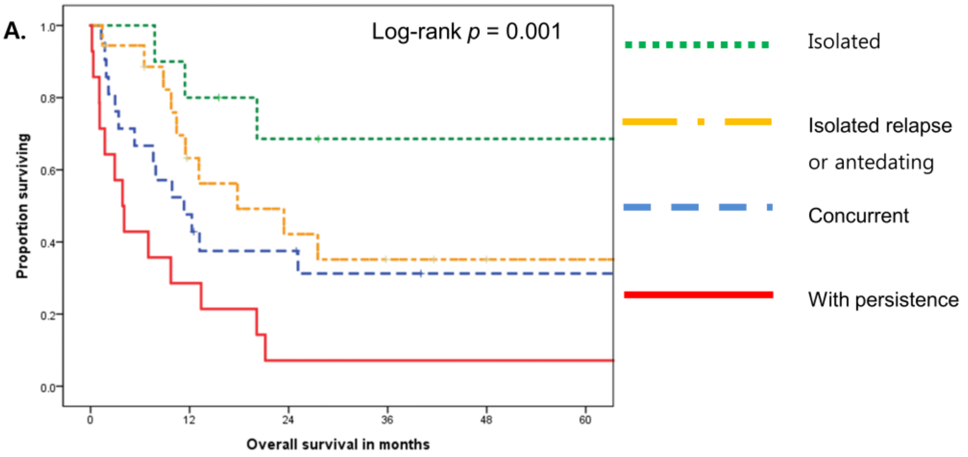

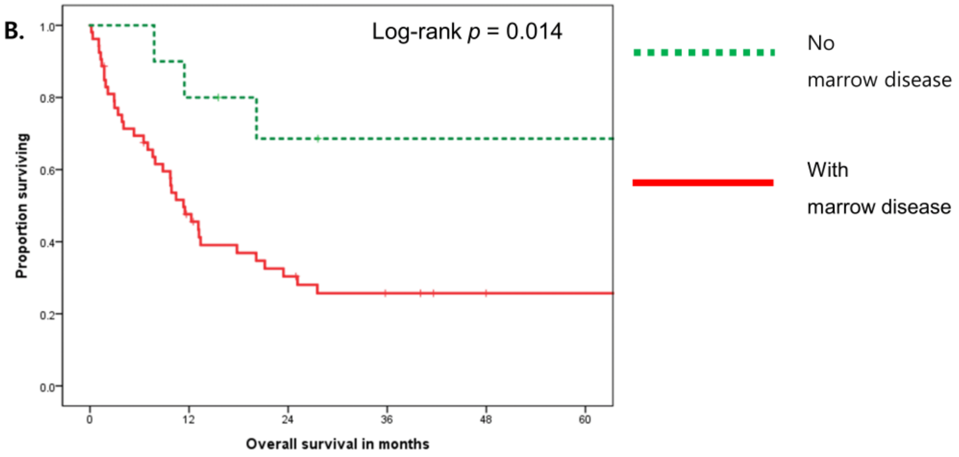

5. References

1. Li H, Durbin R. Fast and accurate short read alignment with Burrows-Wheeler transform. *Bioinformatics* 2009 Jul 15; **25**(14)**:** 1754-1760.

2. DePristo MA, Banks E, Poplin R, Garimella KV, Maguire JR, Hartl C*, et al.* A framework for variation discovery and genotyping using next-generation DNA sequencing data. *Nat Genet* 2011 May; **43**(5)**:** 491-498.

3. Van der Auwera GA, Carneiro MO, Hartl C, Poplin R, Del Angel G, Levy-Moonshine A*, et al.* From FastQ data to high confidence variant calls: the Genome Analysis Toolkit best practices pipeline. *Curr Protoc Bioinformatics* 2013; **43:** 11 10 11-33.

4. Wilm A, Aw PP, Bertrand D, Yeo GH, Ong SH, Wong CH*, et al.* LoFreq: a sequence-quality aware, ultra-sensitive variant caller for uncovering cell-population heterogeneity from high-throughput sequencing datasets. *Nucleic Acids Res* 2012 Dec; **40**(22)**:** 11189-11201.

5. Wei Z, Wang W, Hu P, Lyon GJ, Hakonarson H. SNVer: a statistical tool for variant calling in analysis of pooled or individual next-generation sequencing data. *Nucleic Acids Res* 2011 Oct; **39**(19)**:** e132.
